# Feasibility and efficacy of diabetic retinopathy screening among youth with diabetes in a pediatric endocrinology clinic: a cross-sectional study

**DOI:** 10.1186/s13098-015-0054-z

**Published:** 2015-06-24

**Authors:** Jeffrey L. Tapley, Gerald McGwin, Ambika P. Ashraf, Paul A. MacLennan, Koula Callahan, Karen Searcey, C. Douglas Witherspoon, Jinan Saaddine, Cynthia Owsley

**Affiliations:** Department of Ophthalmology, University of Alabama at Birmingham, Birmingham, AL 35294 USA; Department of Epidemiology, University of Alabama at Birmingham, Birmingham, AL 35294 USA; Department of Pediatrics/Division of Endocrinology, University of Alabama at Birmingham, Birmingham, AL 35294 USA; Department of Surgery, University of Alabama at Birmingham, Birmingham, AL 35294 USA; Vision Health Initiative, Division of Diabetes Translation, Centers for Disease Control and Prevention, Atlanta, GA 30329 USA

**Keywords:** Diabetes mellitus, Diabetes complications, Diabetic retinopathy, Pediatrics, Visual acuity

## Abstract

**Background:**

We examined the feasibility and efficacy of using a non-mydriatic camera to screen for diabetic retinopathy (DR) among youth with type 1 or type 2 diabetes seen in a pediatric endocrinology clinic serving Alabama, the state that has the highest diabetes rate in the United States.

**Methods:**

236 youths with type 1 or type 2 diabetes were screened for DR using a non-mydriatic camera. Visual acuity was also assessed. A questionnaire asked parents about diabetes and eye care history.

**Results:**

Mean duration since diabetes diagnosis was 5.5 years. 66 % reported receiving an eye examination within the previous year. 97.5 % had images that were gradable. DR was detected in 3.8 % of participants. 9.1 % were visually impaired.

**Conclusions:**

Use of a non-mydriatic fundus camera is feasible and efficacious for DR screening in youth with diabetes. DR screening at routine endocrinology visits may be beneficial in managing youth with diabetes and preventing irreversible vision loss, particularly for those in regions where diabetes rates are high.

## Background

Diabetes is a significant, worldwide burden that has dramatically increased in recent years with no evidence of the trend abating. Of particular concern is the rise in both type 1 and type 2 diabetes among youth which has increased by 21.1 % and 30.5 %, respectively, from 2001 to 2009 [1]. As these rates increase, the number of youth with diabetic-related complications will also increase. Among these complications is diabetic retinopathy (DR) and associated vision loss. DR is the leading cause of new cases of blindness among young adults in the United States [2].

It is established that the risk of DR increases with duration of diabetes [3–5]. Retinopathy may not produce apparent symptoms until visual loss develops. Thus, early detection and treatment is important in reducing rates of diabetes related vision problems [6, 7]. However, findings from the SEARCH for Diabetes in Youth study showed that only two-thirds of those under age 18 years with type 1 and type 2 diabetes receive an annual comprehensive dilated eye exam, making it the least commonly followed of the American Diabetes Association (ADA) guidelines for diabetes care in youth [8]. Youth with diabetes were also less likely to obtain annual eye exams than their adult counterparts [9]. Multiple factors may contribute to this low rate of dilated eye examination, such as cost, inconvenience of visiting an eye care provider, transportation challenges, or insufficient knowledge about the risk of DR.

For adults with diabetes, non-mydriatic fundus cameras have been implemented as screening tools for DR [10–12]. These cameras are non-invasive, painless, require minimal time, and produce high-quality fundus images that reveal most clinically significant cases of DR. Those who screen positive can be recommended for a follow-up dilated comprehensive eye examination by an ophthalmologist or optometrist. However, this screening approach has not been widely implemented in youth with diabetes, and few studies have evaluated its feasibility and potential usefulness in pediatric screening for DR [13–15]. The primary objective of this study was to examine the feasibility and efficacy of non-mydriatic diabetic retinopathy screening in youth with type 1 or type 2 diabetes seeking regular diabetes care in a pediatric endocrinology clinic located in Alabama, the state in the US having the highest rate of diabetes [16].

## Methods

This study was conducted at the Pediatric Endocrinology Clinic of the University of Alabama at Birmingham (UAB). This clinic largely serves the state of Alabama, as well as regions of eastern Mississippi and the Florida panhandle. The study population consisted of youth with a clinical diagnosis of either type 1 or type 2 diabetes, ages 8 to 18 years, who attended this clinic. Diabetes diagnosis and type were confirmed by endocrinologists at the clinic. Approval from the Institutional Review Board of UAB was obtained prior to the study’s initiation.

Those meeting the inclusion criteria were identified through the endocrinology clinic’s appointment schedule for the period of June to August 2013. Parents/guardians of eligible youth were mailed a letter describing the study two weeks prior to the youth’s appointment inviting participation at his/her next diabetes clinic visit. At that visit, a research assistant determined if the parent/guardian and youth were interested in participating. Participants were enrolled after obtaining informed consent from the parent/guardian and assent from the youth. A brief survey was administered to the parent/guardian obtaining information on the youth’s history of diabetes, eye care utilization, barriers to receiving eye care, and demographic information. Information was obtained from the medical record on HbA1c, urine microalbumin, and lipid profile (total cholesterol, low-density lipoprotein cholesterol (LDL-C), high-density lipoprotein cholesterol (HDL-C), and triglycerides.

Walk-in letter acuity was assessed for each eye using the TitmusV2 Vision Screener (Sperian Protection Optical, Inc., Chester, VA). Ocular imaging was performed by a trained research assistant using a non-mydriatic fundus camera with autofocus (Model AFC-230, Nidek Inc., Fremont, CA) in a dimly lit room. The youth was positioned in a forehead/chin rest in front of the camera in a seated position. Three images from each eye were taken: the anterior segment of the eye and two images of the fundus (macula centered view and optic nerve head view). If the images were blurry or the youth blinked, additional images were taken to achieve good image quality. Testing lasted about five minutes.

Within one week of screening, the images were reviewed and graded for DR by an ophthalmologist specializing in retinal/vitreal disease using the UK National Health System’s classification system for diabetic retinopathy (Table 1) [17]. The ophthalmologist also noted ocular findings other than DR. Screening results were made available to the endocrinologist, and a letter was generated and sent to the parent/guardian of each participant describing screening results and follow up recommendations. Recommendations were adapted from the American Academy of Ophthalmology’s guidelines for DR follow-up [18]. Participants who screened negative for DR (R0) were sent a letter that encouraged them to make appointments for dilated eye examinations on an annual basis. Participants who screened positive for background DR (R1) or photocoagulation (P) were sent a letter that encouraged follow-up with an eye care provider “in the next few months” for a dilated comprehensive eye exam. Participants with pre-proliferative (R2), proliferative (R3), or maculopathy (M) were sent a letter describing the need for urgent referral to an ophthalmologist. Those who screened positive for any DR (R1, R2, R3, or M) were additionally telephoned by the endocrinologist to describe the results and need for referral. Participants with any of the six images determined unclassifiable/ungradable due to image quality (U) were sent a letter encouraging to follow-up with a comprehensive dilated eye examination.

**Table 1:** Diabetic retinopathy (DR) grading based on the National Health Service Grading Classification System [17] with follow-up recommendations adapted from the American Academy of Ophthalmology [18]

Grade	Description	Recommendation
R0	No diabetic retinopathy	Re-evaluate in twelve months with either eye care provider or photographic screening
R1	Background DR	Refer to eye care provider
R2	Pre-proliferative DR	Refer to ophthalmologist promptly
R3	Proliferative DR	Refer to ophthalmologist promptly
M	Maculopathy	Refer to ophthalmologist promptly
P	Photocoagulation	Refer to eye care provider
U	Unclassifiable/Ungradable	Refer to eye care provider

### Statistical analysis

Participants were categorized into one of three mutually exclusive eye care utilization groups, i.e., ≤12 months, >12 months, and never, based on parent/guardian survey responses regarding their child’s most recent eye care visit. Information on HbA1c and lipid profile (total cholesterol, LDL-C, HDL-C, and triglycerides) was categorized based on gender and age specific norms. Specifically, HbA1c was high if ≥8.5 % (69 mmol/mol) for <6 years of age; ≥8.0 % (64 mmol/mol) for 6–12 years of age; ≥7.5 % (58 mmol/mol) for 13–19 years of age [2]. Lipids were stratified into percentile categories according to published standards [19]. Urine albumin results were classified as normal (<30 mg/L), microalbuminuria (30–299 mg/L), and macroalbuminuria (>300 mg/L) [20]. Eye care utilization categories were compared for significant differences using analysis of variance for continuous variables, and Chi-square tests and Fisher’s exact tests, for categorical variables.

## Results

We identified 575 eligible youths through the appointment schedule and sent letters describing the study. Of these, 252 (44 %) youths were approached in the clinic and invited to participate. The remaining youth were not approached if they were already being seen by the endocrinologist/nurse or were “no-shows” for their appointments; thus, they were unavailable for recruitment. Of the 252 youths approached for participation, 236 (92.9 %) consented to screening. Mean age for participants was 14.1 ± 2.7 years and 135 (57.2 %) were female (Table 2). In terms of race, 67.0 % were white, 29.7 % African American, and the remaining were Hispanic, Native American, or Asian.

**Table 2:** Demographic, clinical, and laboratory characteristics of participants by recency of dilated eye examination

	Total (*N* = 236)	Eye exam ≤ 12 months (*N* = 156)	Eye exam > 12 months (*N* = 47)	Never (*N* = 33)	*P*-value
Demographics					
Age, years, mean (standard deviation, SD)	14.1 (2.69)	14.1 (2.58)	14.9 (2.7)	12.8 (2.8)	0.142
Minimum - maximum	8.0-18.9	8.0-18.9	9.4-18.9	8.3-18.4	
Gender, N (%)					
Male	101 (42.8)	68 (43.6)	22 (46.8)	11 (33.3)	0.459
Female	135 (57.2)	88 (56.4)	25 (53.2)	22 (66.7)	
Race, N (%)					
White, non-Hispanic origin	158 (67.0)	109 (69.9)	25 (59.6)	21 (63.6)	0.225
African American	70 (29.7)	43 (27.6)	17 (36.2)	10 (30.3)	
Hispanic	5 (2.1)	3 (1.9)	0 (0)	2 (6.1)	
Native American	2 (0.9)	1 (0.6)	1 (2.1)	0 (0)	
Asian	1 (0.4)	0 (0)	1 (2.1)	0 (0)	
Clinical characteristics					
Type, n (%)					0.824
Type 1	202 (85.6)	135 (86.6)	39 (83.0)	28 (84.8)	
Type 2	34 (14.4)	21 (13.5)	8 (17.0)	5 (16.0)	
Age at diabetes diagnosis, years, mean (SD)	8.6 (3.9)	8.2 (4.0)	9.6 (4.0)	9.2 (3.5)	0.053
Duration of diabetes diagnosis, years, mean (SD)	5.5 (3.5)	5.9 (3.4)	5.4 (4.0)	3.6 (2.5)	0.001
Laboratory characteristics					
HbA1c high for age^a^, n (%)	169 (71.6)	115 (73.7)	32 (68.1)	22 (66.7)	0.599
Cholesterol^b^, n (%)					
>75th percentile	86 (34.4)	51 (32.7)	26 (55.3)	9 (27.3)	0.009
LDL^b^, n (%)					
>75th percentile	53 (22.5)	33 (21.2)	16 (34.0)	4 (12.1)	0.055
HDL^b^, n (%)					
>75th percentile	191 (80.9)	122 (78.2)	44 (93.6)	25 (75.8)	0.046
Triglycerides^b^, n (%)					
>75th percentile	133 (56.4)	85 (54.5)	33 (70.2)	15 (45.5)	0.064
Urine Albumin^c^ n (%)					0.963
Normal (<30 mg/L)	213 (90.3)	141 (90.4)	42 (89.4)	30 (90.9)	
Microalbuminuria (30–299 mg/L)	22 (9.3)	14 ( 9.0)	5 (10.6)	3 (9.1)	
Macroalbuminuria (≥300 mg/L)	1 (0.4)	1 (0.6)	0 (0)	0 (0)	

^a^HbA1c is high if ≥ 8.5 % (69 mmol/mol) for < 6 years of age; ≥ 8.0 % (64 mmol/mol) for 6 – 12 years of age; ≥ 7.5 % (58 mmol/mol) for 13 – 19 years of age [2]

^b^Percentile categories are based from Gender and Age specific norms [19]

^c^Urine albumin was reported in mg/L; normal was < 30 mg/L, microalbuminuria was defined as ≥ 30 and < 300 mg/L, and macroalbuminuria was defined as ≥ 300 mg/L [20]

Among the study participants, 85.6 % had type 1 diabetes and 14.4 % had type 2 diabetes (Table 2). Mean age at diabetes diagnosis was 8.6 ± 3.9 years, and mean duration of diabetes was 5.5 ± 3.5 years. HbA1c level was “high” in terms of age in 71.6 % of participants. Cholesterol, LDL, HDL, and triglycerides levels were greater than the 75th percentile in 34.4 %, 22.5 %, 80.9 %, and 56.4 % of participants, respectively. Urine albumin was normal (<30 mg/L) in 90.3 % of participants.

66.1 % of participants reported receiving a dilated comprehensive eye examination within the past 12 months, 19.9 % had an examination more than 12 months ago, and 14.0 % had never received an examination (Table 2). Those who had never received an eye examination tended to be younger than those who did have one, although this was not statistically significant (*p* = 0.142). Those who had an eye examination in the past 12 months were more likely to be diagnosed with diabetes at a younger age (*p* = 0.053) and have a longer duration of diabetes (*p* = 0.001). All but one parent/guardian responded that they were capable of taking their child to an ophthalmologist or optometrist to receive a comprehensive dilated eye exam if necessary.

All participants were able to cooperate in sitting before the camera. Images from 6 participants (2.5 %) were determined unreadable (a single eye for 2 and both eyes for 4). All unreadable images were due to the youth’s inability to keep their eyes open during the photographic flash. Mean age for participants with unreadable images was 14.4 years, which was not different than the mean age of participants with readable images (*p* = 0.733).

Nine participants (3.9 %) had DR in at least one eye, the majority of which was background DR (Table 3). One participant with background DR showed signs of maculopathy, and one participant displayed evidence of previous photocoagulation treatment. Ten participants (4.3 %) had other types of ocular findings detected in at least one eye. These included venous loop, media opacities, choroidal coloboma, white centered hemorrhage, pale/abnormal disc, increased cup/disc ratio, and atrophic macular scar. Having any DR (*n* = 9) was associated with duration of diabetes (mean duration 7.8 ± 3.7 years with retinopathy versus 5.4 ± 3.5 years without retinopathy, *p* = 0.046). There were no associations between DR presence and other variables, including age at diagnosis, diabetes type and clinical laboratory values.

**Table 3:** Descriptive characteristics of participants with diabetic eye disease

Finding	Total (*N* = 232)^a^ (%)
Total diabetic eye disease (including DR and maculopathy)	9 (3.9)
Background DR	7 (3.0)
Pre-proliferative DR	0 (0)
Proliferative DR	2 (0.9)
Maculopathy	1 (0.4)
Other lesions	10 (4.3)

^a^Four subjects were unclassifiable/ungradable for both eyes

A sensitivity analysis restricting the study group to those who met ADA guidelines for recommended dilated comprehensive eye examination was performed (i.e., for type 1, a dilated comprehensive eye examination is recommended at the start of puberty or at age ≥10 years, whichever is earlier, once the child has had diabetes for 3–5 years, with annual follow-up thereafter; for type 2, a dilated eye examination is recommended at diagnosis, and then annually thereafter) [2]. This analysis included 140/202 (69.3 %) of participants with type 1 diabetes and all 34 participants with type 2 diabetes. Of the 174 participants who met ADA criteria for dilated examination, two subjects had unclassifiable/ungradable images for both eyes. Among the 172 participants with readable images, eight (4.7 %) subjects had DR in at least one eye, i.e., six (3.5 %) had background DR and two (1.2 %) had proliferative DR.

Overall, 115 participants (49.8 %) had corrective lenses (either glasses or contact lenses); of these, 54 participants (23.4 %) did not bring their corrective lenses to the appointment. Acuity was measured in all participants, even if they did not have their corrective lenses. Table 4 provides acuity screening results for the better and worse seeing eyes; 24.2 % of participants had 20/20 acuity in both eyes; 58.4 % of participants had worse than 20/20 acuity in the better seeing eye, and 75.8 % had worse than 20/20 in the worse seeing eye. 19.5 % of participants met the criteria for visual impairment, defined as 20/40 or worse in the better seeing eye.

**Table 4:** Visual acuity among study participants

Visual acuity	Total sample (*N* = 231)^a^ (%)	Omitting those who had glasses but did not bring them to appointment (*N* = 177)^a^ (%)
Better eye acuity score		
20/20	96 (41.6)	88 (49.7)
20/30	90 (39.0)	73 (41.2)
20/40	29 (12.6)	10 (5.7)
20/50	13 (5.6)	5 (2.8)
20/70	3 (1.3)	1 (0.6)
Worse eye acuity score		
20/20	56 (24.2)	52 (29.4)
20/30	92 (39.8)	81 (45.8)
20/40	38 (16.5)	26 (14.7)
20/50	26 (11.3)	10 (5.7)
20/70	11 (4.8)	5 (2.8)
20/100	8 (3.5)	3 (1.7)

^a^Five participants were unable to perform visual acuity test

Because almost one fourth of the sample had corrective lenses but did not bring them, results could be influenced by a refractive error that is normally corrected. When Table 4 excluded participants who did not bring their lenses, only 29.4 % had 20/20 acuity in both eyes. After excluding participants who did not bring their glasses, acuity worse than 20/20 was associated with a younger age (*p* = 0.042), a shorter duration of diabetes (*p* = 0.030), and type 2 diabetes (*p* = 0.015); 32.3 % of participants with type 1 diabetes and 5.3 % with type 2 diabetes had 20/20 acuity in both eyes. Visual acuity did not differ with regard to gender, race/ethnicity, recency of last dilated eye exam, age at diabetes diagnosis, or presence of DR.

## Discussion

Our results suggest that non-mydriatic fundus imaging among youth with diabetes conducted at a routine endocrinology appointment is a feasible way to screen for DR. Almost all parents/guardians who were approached agreed to have their son or daughter screened, and over 97 % of participants had images that were gradable, a rate that is comparable to other pediatric DR screening studies using a non-mydriatic camera [13, 14]. These rates of readable images are considerably higher than adult rates [21, 22], which may stem from the larger pupil size typical of youth. Increasing age is the strongest predictor of ungradable image rates for non-mydriatic imaging [23]. Good imaging quality was just as likely in younger versus older youth. The average time for screening was brief, considerably less time than required for a dilated eye examination. The brevity of the screening process and the reduced burden of not dilating the pupil may facilitate increasing the number of youth screened for DR.

A small percentage of youth screened positive for DR (~4 %), not surprising since pediatric populations typically have a briefer duration of diabetes compared to adults, which decreases the risk for DR [3–5]. Given that 33 % of participants in this study had not received a dilated eye examination in the past year or never had one, the endocrinologist office screening represented a convenient opportunity to assess retinal health. The vast majority of DR detected was background DR (7 of 9 cases), which suggests high potential for intervention in DR’s earliest phases when treatment can prevent vision loss [2].

Only 66 % of participants received a dilated eye exam within the previous 12 months, which agrees with the rate reported in the SEARCH for Diabetes in Youth study [8, 9]. Even lower rates have been reported in studies based in Philadelphia (64 %) and Ohio (53.5 %) [24, 25]. Low rates of annual eye care utilization is concerning since early DR detection and treatment reduce the risk of irreversible vision loss [2]. Our results indicate that youth with a longer duration of diabetes were more likely to receive an eye exam within the past year, agreeing with a recent study [14]. Although this trend is desirable, it is still apparent that many youth with diabetes underutilize eye care services.

All but one parent/guardian in our study stated they were capable of taking their child to an eye care provider to receive a comprehensive eye care if necessary, and did not cite barriers to care. This suggests that efforts to improve dilated eye examination rates should focus on educating parents/guardians about the importance of routine eye care for youth with diabetes. Another potential mechanism underlying low dilated examination rates among youth with diabetes may be that pediatricians and/or endocrinologists are not effectively communicating the importance of annual dilated eye care during routine patient check-ups. Research has shown that adults with diabetes are more compliant to vision guidelines when screening is routinely recommended by their physician [26]. However, research on teenagers found that only 35 % of teenagers with diabetes were referred by their endocrinologist to eye care providers for annual examinations [27]. Thus, it appears that pediatricians, other primary care physicians, and endocrinologists could be more vigilant in recommending annual dilated eye examinations for their patients with diabetes.

One important aspect of any screening program is its potential to reduce health care costs, including the financial burden of untreated disease [28]. There is growing evidence that non-mydriatic cameras are a cost-effective screening tool for DR in adults with diabetes [29]; however, there has been little research on the cost-effectiveness of DR screening tools for youth with diabetes. Huo et al. [30] found that implementing ADA recommendations for annual dilated exams were not cost effective for youth with type 1 diabetes who had excellent glycemic control, given the low rates of retinopathy actually present in these patients. Non-mydriatic cameras in the endocrinology office may be a cost-effective alternative tool for pediatric DR detection.

Most studies show a direct correlation between duration of disease and development of DR, with retinopathy occurring after at least 3 years since diagnosis and typical duration of diabetes greater than 5 years [2, 31]. In this study, we report a mean duration of 7.8 years for those with retinopathy compared to 5.4 years without retinopathy (*p* = 0.0461). Interestingly, we identified two cases of background retinopathy present after only 2 years since diagnosis – one case each in both a type 1 and type 2 youth with diabetes. Other studies have also reported early retinopathy in youth with less than three years duration [32, 33]. This is notable, considering current ADA guidelines for type 1 diabetes recommend screening to begin for those ≥ 10 years old having diabetes for 3–5 years [2]. With regard to clinical characteristics, our study did not identify any significant risk factors associated with retinopathy other than duration of diabetes. Further studies are needed to better identify clinical variables and their associations with DR in order to provide more effective and targeted screening in youth with diabetes [34].

There has been little research on vision impairment among youth with diabetes. In our study, almost half (49.8 %) of the sample used corrective lenses. However, only 47 % of those with corrective lenses wore them to their regular endocrinologist appointment at the time of screening, suggesting that some youth with diabetes are not wearing corrective lenses routinely even though they have been prescribed. Further exploration may provide insights into reasons for spectacle noncompliance in youth with diabetes. Walk-in visual acuity for many youth in our sample was markedly low. Even after excluding those who did not bring their corrective lenses to the screening, 9.1 % of participants met the criteria for visual impairment and only 29.4 % had 20/20 vision in both eyes. As most parents/guardians of youth with diabetes understand the importance of routine visits to the endocrinologist and/or pediatrician, there appears to be room for improved education on the importance of vision care.

Strengths and limitations of this study should be noted. To our knowledge, this is the only study that has examined the feasibility of DR screening in youth using a non-mydriatic camera during routine pediatric endocrinology clinic visits. The pediatric endocrinology clinic serving as the study setting is in Alabama, which has the highest rate of diabetes in the US [16]. Although the study was unable to enroll all youth scheduled for an appointment during the study period, nearly all parents/guardians who were invited consented to participate. A limitation was the self-reported diabetes history and vision care utilization obtained from our survey, rather than extracting this information from medical records. The extent to which participants who screened positive for DR or other ocular findings pursued follow-up examinations with an ophthalmologist or optometrist was not part of the study’s scope, but this issue will be addressed in future work. Future work should also compare the non-mydriatic photography method used in this study to the clinical gold-standard dilated eye examination.

## Conclusion

We have shown that a non-mydriatic fundus camera is a feasible and efficacious method to screen for diabetic retinopathy in youth with diabetes in a region of the US that has the highest rate of diabetes in the nation. With approximately 1/3 of youth with diabetes not receiving annual, dilated eye exams, screening programs implemented during a routine endocrinologist appointment may be beneficial in the management of youth with diabetes and prevention of irreversible vision loss, particularly in those regions of the US with high diabetes rates.

## Abbreviations

ADA: American diabetes association
DR: Diabetic retinopathy
HDL-C: High-density lipoprotein cholesterol
LDL-C: Low-density lipoprotein cholesterol
UAB: University of Alabama at Birmingham
